# Associations between urbanicity and malaria at local scales in Uganda

**DOI:** 10.1186/s12936-015-0865-2

**Published:** 2015-09-29

**Authors:** Simon P. Kigozi, Deepa K. Pindolia, David L. Smith, Emmanuel Arinaitwe, Agaba Katureebe, Maxwell Kilama, Joaniter Nankabirwa, Steve W. Lindsay, Sarah G. Staedke, Grant Dorsey, Moses R. Kamya, Andrew J. Tatem

**Affiliations:** Infectious Diseases Research Collaboration, Kampala, Uganda; Clinton Health Access Initiative, Boston, USA; Fogarty International Center, National Institutes of Health, Bethesda, USA; Department of Zoology, University of Oxford, Oxford, UK; Sanaria Institute for Global Health and Tropical Medicine, Rockville, MD USA; School of Biological and Biomedical Sciences, Durham University, Durham, UK; London School of Hygiene and Tropical Medicine, London, UK; Department of Medicine, San Francisco General Hospital, University of California, San Francisco, CA USA; School of Medicine, Makerere University College of Health Sciences, Kampala, Uganda; Department of Geography and Environment, University of Southampton, Southampton, UK; Flowminder Foundation, Stockholm, Sweden

**Keywords:** Urbanization, *Plasmodium falciparum*, Remote sensing, GIS, Urban malaria

## Abstract

**Background:**

Sub-Saharan Africa is expected to show the greatest rates of urbanization over the next 50 years. Urbanization has shown a substantial impact in reducing malaria transmission due to multiple factors, including unfavourable habitats for *Anopheles* mosquitoes, generally healthier human populations, better access to healthcare, and higher housing standards. Statistical relationships have been explored at global and local scales, but generally only examining the effects of urbanization on single malaria metrics. In this study, associations between multiple measures of urbanization and a variety of malaria metrics were estimated at local scales.

**Methods:**

Cohorts of children and adults from 100 households across each of three contrasting sub-counties of Uganda (Walukuba, Nagongera and Kihihi) were followed for 24 months. Measures of urbanicity included density of surrounding households, vegetation index, satellite-derived night-time lights, land cover, and a composite urbanicity score. Malaria metrics included the household density of mosquitoes (number of female *Anopheles* mosquitoes captured), parasite prevalence and malaria incidence. Associations between measures of urbanicity and malaria metrics were made using negative binomial and logistic regression models.

**Results:**

One site (Walukuba) had significantly higher urbanicity measures compared to the two rural sites. In Walukuba, all individual measures of higher urbanicity were significantly associated with a lower household density of mosquitoes. The higher composite urbanicity score in Walukuba was also associated with a lower household density of mosquitoes (incidence rate ratio = 0.28, 95 % CI 0.17–0.48, p < 0.001) and a lower parasite prevalence (odds ratio, OR = 0.44, CI 0.20–0.97, p = 0.04). In one rural site (Kihihi), only a higher density of surrounding households was associated with a lower parasite prevalence (OR = 0.15, CI 0.07–0.34, p < 0.001). And, in only one rural site (Nagongera) was living where NDVI ≤0.45 associated with higher incidence of malaria (IRR = 1.35, CI 1.35–1.70, p = 0.01).

**Conclusions:**

Urbanicity has been shown previously to lead to a reduction in malaria transmission at large spatial scales. At finer scales, individual household measures of higher urbanicity were associated with lower mosquito densities and parasite prevalence only in the site that was generally characterized as being urban. The approaches outlined here can help better characterize urbanicity at the household level and improve targeting of control interventions.

## Background

Urbanization has generally been associated with a reduction in malaria transmission across spatial scales [1–5]. Lower transmission of both *Plasmodium falciparum* [5] and *Plasmodium vivax* [3] has been found consistently in urban areas, compared to surrounding areas and multiple factors contribute to this. Urban areas generally have fewer mosquito breeding sites, better access to treatment and higher intervention coverage levels than rural areas [6, 7]. Urbanization also involves significant ecological change and socio-economic change, including improved health, housing and wealth factors that impel significant entomological, parasitological and behavioural effects that generally reduce malaria transmission [2, 4, 5] and many malaria-endemic nations have recorded reduced malaria transmission in major cities [2, 8]. Previous studies on the impact of ‘urbanicity’ have generally looked at malaria parasite prevalence over large scales or at specific metrics at small scales, and using a single indicator of urbanicity [9]. No studies have examined a full range of entomological, parasitological and clinical-based metrics across multiple transmission sites and using multiple metrics of urbanicity.

Malaria remains a major problem in Uganda and many efforts have been made to control it including, but not limited to, provision and/or promotion of long-lasting insecticidal nets (LLINs) and indoor residual spraying of insecticides (IRS) [10]. Interventions to date have predominantly targeted vulnerable populations such as expectant mothers and children, and been implemented uniformly across wide geographic areas. However, approaches that target interventions to local conditions could improve efficiency and impact in settings where resources are limited. Aside from the targeted implementation of interventions, there is a need to better predict the effects that trends in urbanization, climate change, land use and development are likely to have on the burden of malaria in Uganda and other African countries. Most of Uganda’s population live in relatively high-transmission areas [11] and despite recent expansion of control efforts, evidence of a clear reduction in the burden of disease is lacking [12, 13]. A better understanding of the relationship between urbanicity and malaria risks at various scales may help to improve the targeting of strategies for malaria control, such as the initiation of integrated vector management (IVM) where larval source management (LSM) may be the chosen approach for urban locales, and IRS the approach for the rural, less densely populated areas [14]. Urbanization is a highly relevant topic for planning in Uganda because, although the populations are currently predominantly rural, high rates of population growth are leading to rapid urbanization.

Here, existing cohort studies were utilized in an exploratory analysis to examine associations between various measures of urbanicity and entomological, parasitological and clinical malaria metrics. The studies were conducted in three sites in Uganda with different levels of urbanicity and transmission intensity. Because of the underlying differences in the sites, several physical and infrastructure-based urbanicity metrics were evaluated that had consistent and quantifiable spatial characteristics, including the densities of surrounding households from survey data, and satellite image variables covering land use, night-time lights and occurrence or amount of vegetation.

## Methods

### Study sites

**Fig. 1:**
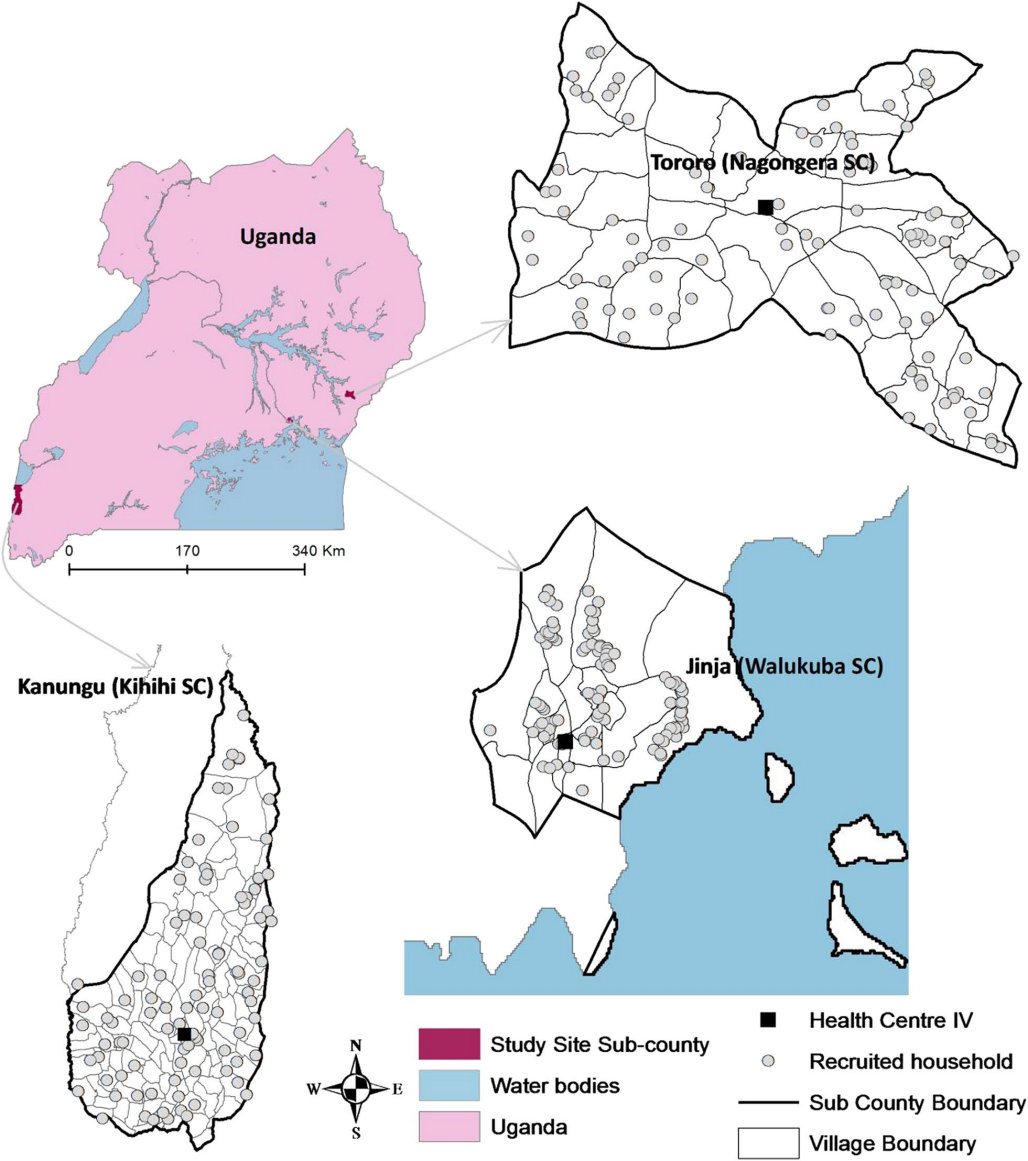
Map of Uganda showing the location of the study sites: three sub-counties including the cohort households (100 per site) as well as health centres where participants were attended to. Entomological measures were taken at these same households

Cohort studies were conducted at three sites in Uganda:(1) Walukuba sub-county in Jinja district ≈12.9 sq km (00°26′33·2″N, 33°13′32·3″E) at the shores of Lake Victoria; (2) Kihihi sub-county in Kanungu district ≈186 sq km (00°45′03·1″S, 29°42′03·6″E) in the southwest part of Uganda; and, (3) Nagongera sub-county in Tororo district ≈81 sq km (00°46′10·6″N, 34°01′34·1″E) close to the Uganda–Kenya border. Figure 1 shows the location of the sites. Clinical activities were conducted at the respective health sub-district level IV health centres.

Jinja district was historically the industrial capital of Uganda and as such, Walukuba was urbanized in the industrial times of 1950–80s, hence the presence of factory buildings (now deserted for the most part), factory worker estates, more tarmac roads, and wider coverage of hydro-electric power than other sites. In Nagongera, according to a 2009/2010 survey, 2.6 % of households had electricity, 59.8 % of the homes were grass thatched [15], and less than half a kilometre of tarmac road was observed. Kihihi has similar characteristics to Nagongera, with almost no tarmac road (less than 3 km), similar housing structures (walls of mud and poles) and its sources of water predominantly being open wells, bore holes and rainwater harvesting.

### Participant recruitment and follow-up

All households in the three sub-counties were enumerated in a census survey and geo-located using a Garmin e-Trex Legend H GPS unit (Garmin International, Inc. Olathe, KS, USA). A household was defined as any single permanent or semi-permanent dwelling structure for humans who generally cook and/or eat together. One hundred households were randomly selected from each of the sub-counties and all children aged 6 months to 10 years and one adult primary caregiver from each household were enrolled as previously described [16]. At enrolment a survey was performed for each household. Figure 1 shows the distribution of households that were included in the cohort studies.

The cohorts were followed from August 2011 to September 2013, and they were dynamic, such that all newly eligible children were enrolled and study participants who reached 11 years of age were excluded from further follow-up. At enrolment, study participants and their parents/guardians were given a LLIN and underwent a standardized evaluation, including a history, physical examination and collection of blood for haemoglobin measurement and thick/thin blood smear. Cohort study participants received all medical care free of charge at a designated study clinic open every day. Participants who presented with a documented fever (tympanic temperature >38.0 °C) or history of fever in the previous 24 h had blood obtained by finger prick for a thick blood smear. If the smear had malaria parasites, the patient was diagnosed with malaria. Episodes of uncomplicated malaria were treated with artemether–lumefantrine and episodes of complicated malaria or recurrent malaria occurring within 14 days of prior therapy were treated with quinine. Routine thick blood smears were done every 3 months to estimate parasite prevalence.

Entomological surveys were conducted monthly from August 2011 to September 2013 in each household using miniature CDC light traps (Model 512; John W. Hock Company, Gainesville, FL, USA) with the light positioned 1 m above the floor at the foot end of the bed where a cohort study participant slept. Traps were set at 19.00 h and collected at 07.00 the following morning by field workers. Methods used for the processing of mosquito specimens were as described previously [17].

### Malaria metrics

Malaria metrics used in this study included the household density of mosquitoes (HDM), parasite prevalence by light microscopy, and the incidence of malaria. The HDM was calculated as the total number of female *Anopheles* mosquitoes captured divided by the number of house-nights of collection. Parasite prevalence was calculated as the number of routine blood smears positive for asexual parasites divided by the total number of routine blood smears done. The incidence of malaria was calculated as the number of episodes of malaria divided by the person years of observation. The malaria metrics used here can be placed on a spectrum that is comprised of three major components: the transmission phase, at the frontline between environment and the human host; the infection phase, an indication of malaria parasites independent of symptoms; and, the clinical phase, which manifests itself as a documented infection in the setting of symptomatic illness. In this study, HDM is in the first phase of this spectrum while parasite prevalence is apportioned to the second phase, one level removed from the environment, and the incidence of malaria is apportioned to the third phase, two levels removed from the environment.

### Measures of urbanicity

Many different methods have been used in previous studies to provide consistent measures of either binary urban–rural classifications, or continuous quantification of urbanicity. Urbanicity can be measured in multiple ways and in this study physical-based measures that could be consistently derived between and across sites were measured, including household density, land cover, vegetation amount, and night-time light brightness. For each cohort household, 100-m buffer zones, also known as observational buffers, were created for generating estimates of household level measures of urbanicity by extracting data using ESRI ArcGIS 10.1 software (ESRI 1995–2013; Redlands, CA, USA). Cohort households accounted for 0.8, 1.0 and 1.4 % of the total number of households enumerated in the Kihihi, Walukuba, and Nagongera sub-counties, respectively.

#### Household density

Household density was defined as the number of households enumerated in the observational buffers around each cohort study household.

#### Land cover

**Fig. 2:**
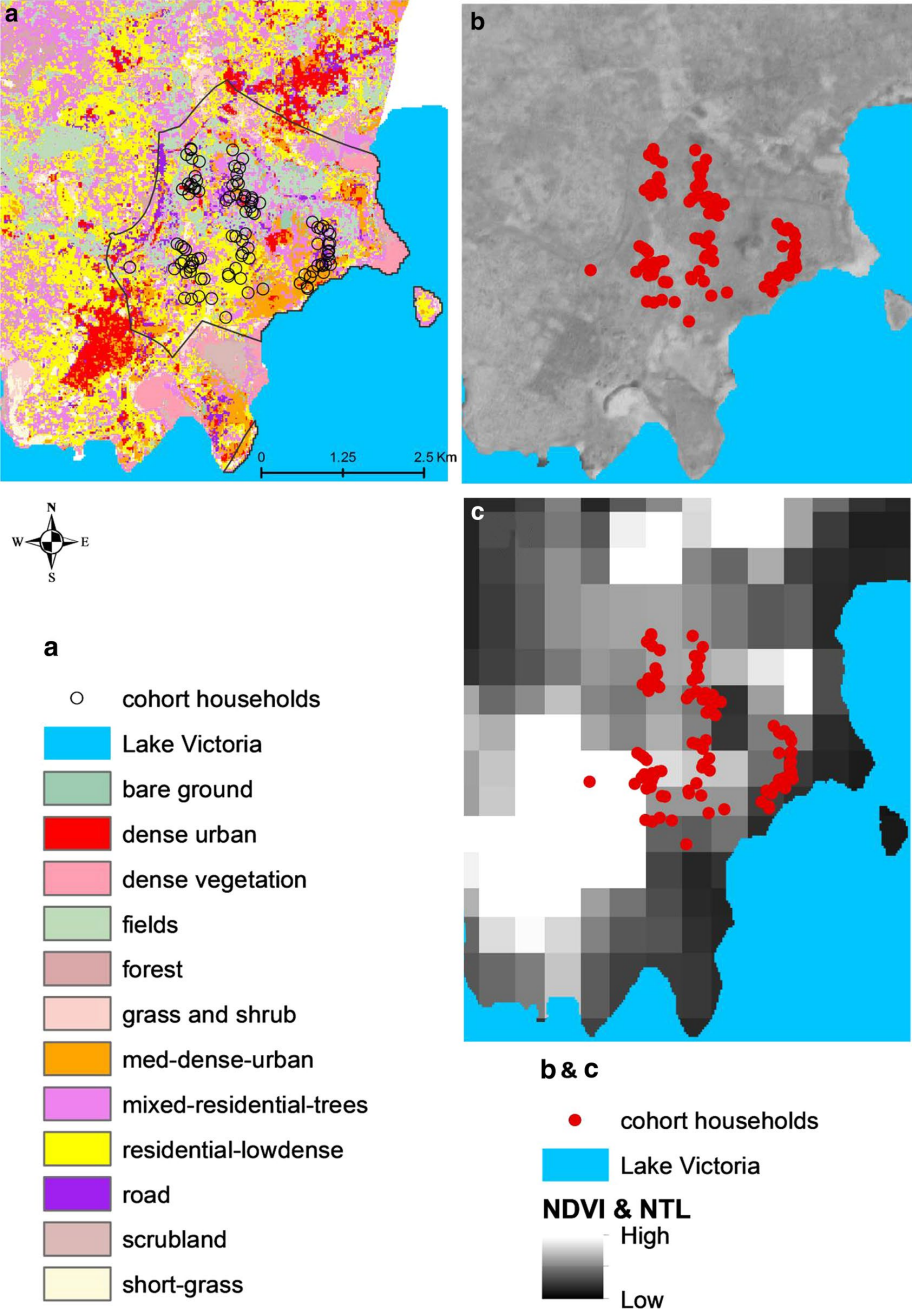
Distribution of three urbanicity metrics across the three sites, based on the frequency distribution of individual metric values within a 100 m buffer around households participating in the cohort and entomology study in each site. **a** Land cover classification in Walukuba at 30 m spatial resolution overlaid with the participating study households. **b** NDVI in Walukuba at 6 m spatial resolution. **c** Satellite-derived night-time light brightness across Walukuba overlaid with the participating study households

A supervised classification of Landsat imagery was undertaken, using Google earth [18] to define training samples of known land cover type. Landsat Enhanced Thematic Mapped (ETM) images matching the period of data collection were obtained from the US geological survey repository [19]. Following previously defined approaches [20], the training samples were used within a maximum likelihood supervised classifier in the satellite image processing software, Erdas Imagine (Geosystems, L. 2004; ERDAS imagine. Atlanta, GA, USA), to produce a land cover map that contained classes representing different gradations of urbanicity (Fig. 2). Among the three sites, only Walukuba displayed distinctly observable urban classes. The others, Nagongera and Kihihi, did not display similar classes, and thus, classification results were not used here for those sites. Within Walukuba, three distinct ‘urban’ related classes existed: dense urban, medium dense urban and residential low dense urban (Fig. 2). The percentage of 30 × 30-m grid cells in the observational buffer in each of these classes was used as a household level metric of urbanicity to test against the malaria metrics. Only the residential low dense urban classification of land cover showed significant associations with malaria metrics and hence was maintained while the other two were dropped from further analysis.

#### Normalized difference vegetation index (NDVI)

NDVI is measured on a scale of −1 to 1 where the extreme negative values represent water; values around zero reflect barren earth surfaces such as rock, sand or snow; medium positive values represent bushes or grassland; and, the higher positive values (≥0.6) represent temperate/tropical rainforest cover [21]. NDVI often forms the basis for mapping degrees of urbanicity in well-vegetated regions of the world, where urbanized regions are relatively lowly vegetated compared to surrounding rural areas. In this study, SPOT 6, 6-m resolution satellite imagery acquired over 2013 with at most 1 % cloud cover (Astrium GEO-Information Services, 2013, South Africa) was used to generate NDVI.

#### Night-time lights

Urban areas generally display greater rates of electrification than rural ones [22, 23], and thereby the amount of lighting detected through night-time satellite imagery has been used to quantify both the presence of urban areas and degree of urbanicity [2, 3]. In this study, imagery from the visible infrared imaging radiometer suite (VIIRS) satellite sensor was obtained for the three sites (from http://ngdc.noaa.gov/eog/viirs) and the levels of brightness within the observational buffers were estimated on a relative scale.

#### Composite urbanicity score

In addition to the individual urbanicity metrics measured in this study, overall urbanicity at each participating household, referred to as the composite urbanicity score or composite score, was estimated using the values of the individual urbanicity metrics in the observational buffer of the household. A score of 1 was assigned for every instance of a high level of individual urbanicity metric and 0 otherwise (Tables 1, 2, 3), and the cumulative score, between 0 and 4, from the four individual urbanicity metrics was assigned as the composite score for the participating household.

**Table 1:** Associations between measures of urbanicity and the household density of mosquitoes stratified by study site

Urbanicity metric	Exposure categories	Walukuba			Kihihi			Nagongera		
		HDM (nights of collection)^a^	IRR^b^ (95 % CI)	P	HDM (nights of collection)^a^	IRR^b^ (95 % CI)	P	HDM (nights of collection)^a^	IRR^b^ (95 % CI)	P
Household density^c^	≤80	2.08 (327)	0.30 (0.16–0.59)	<0.001	4.68 (2086)	0.68 (0.28–1.64)	0.39	43.3 (2329)	N/A	
	>80	0.90 (1857)			3.58 (166)			None		
NDVI^d^	>0.45	1.85 (707)	0.35 (0.21–0.57)	<0.001	4.78 (1704)	0.83 (0.48–1.42)	0.49	41.1 (1660)	1.16 (0.87–1.54)	0.32
	≤0.45	0.71 (1477)			4.04 (548)			48.7 (669)		
Night-time lights	≤3	1.33 (1526)	0.32 (0.19–0.55)	<0.001	4.60 (2252)	N/A		43.3 (2329)	N/A	
	>3	0.49 (658)			None			None		
Land cover	≤20 %	1.37 (1265)	0.42 (0.26–0.69)	0.001	Not measured			Not measured		
	>20 %	0.68 (919)								
Composite score^e^	Low	1.37 (1501)	0.28 (0.17–0.48)	<0.001	4.68 (2086)	0.68 (0.28–1.64)	0.39	43.3 (2329)	N/A	
	High	0.44 (683)			3.58 (166)			None		

^a^Household density of mosquitoes (number of adult female anophelines caught per nights of collection)

^b^Incidence rate ratio

^c^Number of households within 100 m radius from participating household

^d^Normalized difference vegetation index

^e^1 point for each individual urbanicity metric: Walukuba (low = 0–2, high = 3–4), Kihihi and Nagongera (low = 0–1, high = 2)

**Table 2:** Associations between measures of urbanicity and parasite prevalence stratified by study site

Urbanicity metric	Exposure categories	Walukuba			Kihihi			Nagongera		
		PP^a^ (total blood smears)	OR^b^ (95 % CI)	P	PP (total blood smears)	OR^b^ (95 % CI)	P	PP (total blood smears)	OR^b^ (95 % CI)	P
Household density^c^	≤80	7.9 % (406)	0.83 (0.33–2.11)	0.70	8.4 % (3151)	0.15 (0.07–0.34)	<0.001	22.5 % (3231)	N/A	
	>80	5.8 % (2207)			1.3 % (225)			None		
NDVI^d^	>0.45	7.3 % (854)	0.67 (0.33–1.34)	0.26	8.3 % (2563)	0.76 (0.34–1.67)	0.49	23.4 % (2247)	0.87 (0.63–1.20)	0.40
	≤0.45	5.5 % (1759)			6.5 % (813)			20.4 (984)		
Night-time lights	≤3	6.8 % (1799)	0.72 (0.34–1.51)	0.38	7.9 % (3376)	N/A		22.5 % (3231)	N/A	
	>3	4.6 % (814)			None			None		
Land cover	≤20 %	6.8 % (1496)	0.84 (0.41–1.71)	0.63	Not measured			Not measured		
	>20 %	5.2 % (1117)								
Composite score^e^	Low	7.3 % (1808)	0.44 (0.20–0.97)	0.04	8.4 % (3151)	0.15 (0.07–0.34)	<0.001	22.5 % (3231)	N/A	
	High	3.4 % (805)			1.3 % (225)			None		

^a^Parasite prevalence: proportion of blood smears positive for asexual parasites

^b^Odds ratio adjusted for age at the time of the blood smear and repeated measures in the same household

^c^Number of households within 100 m radius from participating household

^d^Normalized Difference Vegetation Index

^e^1 point for each individual urbanicity metric: Walukuba (low = 0–2, high = 3–4), Kihihi and Nagongera (low = 0–1, high = 2)

**Table 3:** Associations between measures of urbanicity and incidence of malaria stratified by study site

Urbanicity metric	Exposure categories	Walukuba			Kihihi			Nagongera		
		Malaria incidence (PY)^a^	IRR^b^ (95 % CI)	P	Malaria incidence (PY)^a^	IRR^b^ (95 % CI)	P	Malaria incidence (PY)^a^	IRR^b^ (95 % CI)	P
Household density^c^	≤80	0.35 (94.2)	1.02 (0.58–1.81)	0.94	1.21 (720.2)	0.48 (0.20–1.17)	0.11	2.17 (746.9)	N/A	
	>80	0.36 (512.5)			0.60 (51.6)			None		
NDVI^d^	>0.45	0.43 (197.6)	0.77 (0.46–1.28)	0.31	1.19 (586.4)	0.97 (0.64–1.45)	0.87	1.91 (519.6)	1.35 (1.07–1.70)	0.01
	≤0.45	0.32 (409.1)			1.11 (185.3)			2.75 (227.3)		
Night-time lights	≤3	0.36 (418.3)	1.04 (0.62–1.75)	0.88	1.17 (771.8)	N/A		2.17 (746.9)	N/A	
	>3	0.36 (188.4)			None			None		
Land cover	≤20 %	0.37 (346.6)	1.04 (0.62–1.73)	0.89	Not measured			Not measured		
	>20 %	0.35 (260.1)								
Composite score^e^	Low	0.37 (419.0)	0.97 (0.57–1.65)	0.92	1.21 (720.2)	0.48 (0.20–1.17)	0.11	2.17 (746.9)	N/A	
	High	0.34 (187.7)			0.60 (51.6)			None		

^a^Number of episodes of malaria per person years (PY) of follow-up

^b^Incidence rate ratio adjusted for mean age during follow-up and repeated measures in the same household

^c^Number of households within 100 m radius from a participating household

^d^Normalized difference vegetation index

^e^1 point for each individual urbanicity metric: Walukuba (low = 0–2, high = 3–4), Kihihi and Nagongera (low = 0–1, high = 2)

### Statistical analyses

Data collected were analysed using Stata 12 (StataCorp, TX, USA). Lowess smoothing plots were used to explore the relationships between continuous measures of urbanicity and the various malaria metrics. Based on these exploratory analyses, continuous measures of urbanicity for participating households were dichotomized into the following categories for estimating associations with malaria metrics: (1) household density (≤80 vs >80 households per 100-m radius buffer); (2) night-time lights (≤3.0 vs >3.0 units of single band 32 bit pixels); (3) percentage residential low dense urban land cover (≤20 vs >20 %); (4) NDVI (≤0.45 vs >0.45); (5) composite score (low versus high, see Tables 1, 2, 3). At the household level, negative binomial regression was used to model the relationships between measures of urbanicity and the HDM (number of female *Anopheles* caught per house), with the number of sampling nights included as an offset term in the model. At the individual cohort participant level, logistic regression was used to model the relationships between measures of urbanicity and the odds of malaria infection (parasite prevalence) at the time of each routine clinic visit, and negative binomial regression was used to model these relationships with the incidence of malaria, with the duration of follow-up included as an offset term. Analyses at the individual level were adjusted for age of the study participants and robust standard errors were used to adjust for clustering of cohort participants living in the same household. Associations using negative binomial regression models were expressed as the incidence rate ratio (IRR) and associations using logistic regression models were expressed as the odds ratio (OR). A p value <0.05 was considered statistically significant.

### Ethical approval

Ethical approval was obtained from the Makerere University School of Medicine Research and Ethics Committee, the Uganda National Council for Science and Technology, the London School of Hygiene and Tropical Medicine Ethics Committee, the School of Biological and Biomedical Sciences Ethics Committee, Durham University and the University of California, San Francisco Committee on Human Research.

## Results

### Study population

A total of 1167 participants (878 children and 289 adults) in 300 households were enrolled and followed over the 2-year period; 348 in Walukuba, 419 in Kihihi and 400 in Nagongera. Overall, 1087 participants (93.1 %) and 292 households (97.3 %) were followed for at least 6 months. Characteristics of study households and cohort participants are presented in Table 4. Households in Walukuba were much more characteristic of an urban environment compared to the two rural sites (Kihihi and Nagongera), including a higher proportion with electricity (22 vs 3 %, p < 0.001) and the use of charcoal versus firewood for cooking (82 vs 5 %, p < 0.001), a higher density of surrounding households (225 vs 13 within a 100 m radius, p < 0.001), a lower vegetation index (66 vs 27 % with NDVI <0.45 units, p < 0.001), and more night-time lights (29 vs 0 % with >3 units, p < 0.001). In addition, the number of female *Anopheles* mosquitoes captured per night was significantly lower in Walukuba (HDM = 1.1) compared to Kihihi (HDM = 4.6, p < 0.001) and Nagongera (HDM = 43.3, p < 0.001). At the level of the individual cohort participants, parasite prevalence in Walukuba (6.1 %) was similar to Kihihi (7.9 %, p = 0.14) but significantly lower than in Nagongera (22.5 %, p < 0.001). The incidence of malaria in Walukuba (0.36 episodes per person year (PY)) was significantly lower than in Kihihi (1.17 episodes PY, p < 0.001) and Nagongera (2.17 episodes PY, p < 0.001).

**Table 4:** Characteristics of study households and participants stratified by study site

Characteristics	Walukuba	Kihihi	Nagongera
At the household level			
Number of households	100	100	100
Average altitude (m)	1165	1103	1130
Electricity in home	22 %	4 %	1 %
Uses charcoal for cooking	82 %	10 %	0 %
Density of surrounding households^a^, mean (SD)	225 (152)	19 (32)	7 (6)
NDVI <0.45 units^a^	66 %	26 %	28 %
Night-time lights >3 units^a^	29 %	0 %	0 %
Mean land cover >20 % residential urban^a^	41 %	N/A	N/A
Total number of nights of mosquito collections	2184	2252	2329
Total number of female *Anopheles* mosquitoes	2358	10,370	100,890
Household density of mosquitoes per night (95 % CI)	1.1 (1.0–1.1)	4.6 (4.5–4.7)	43.3 (43.1–43.6)
At the individual cohort participant level			
Number of participants	348	419	400
Mean age in years during follow-up (children)	5.0	5.6	5.4
Mean age in years during follow-up (adults)	33.4	38.3	39.0
Total number of routine blood slides	2613	3376	3231
Parasite prevalence (95 % CI)	6.1 (5.2–7.1 %)	7.9 (7.0–8.8 %)	22.5 (21.0–23.9 %)
Person years of observation	606.7	771.8	746.9
Total episodes of malaria	217	905	1619
Incidence of malaria per person years (95 % CI)	0.36 (0.31–0.41)	1.17 (1.10–1.25)	2.17 (2.06–2.28)

^a^Within 100 m radius of study household

### Mosquito species composition

The totals of female *Anopheles* collected over the 24-month period were 2358 in Walukuba, 10,370 in Kihihi and 100,797 in Nagongera. The most common species was *Anopheles gambiae*, with 86.3 % of those caught in Walukuba, 99.0 % in Kihihi and 90.3 % in Nagongera. *Anopheles funestus*, made up 6.7 % of catches in Walukuba, 0.0 % in Kanungu and 9.1 % in Nagongera. Finally, other female *Anopheles* species caught together made up 7.0 % of catches in Walukuba, 1.0 % in Kihihi and 0.7 % in Nagongera.

### Associations between measures of urbanicity and the household density of mosquitoes

**Fig. 3:**
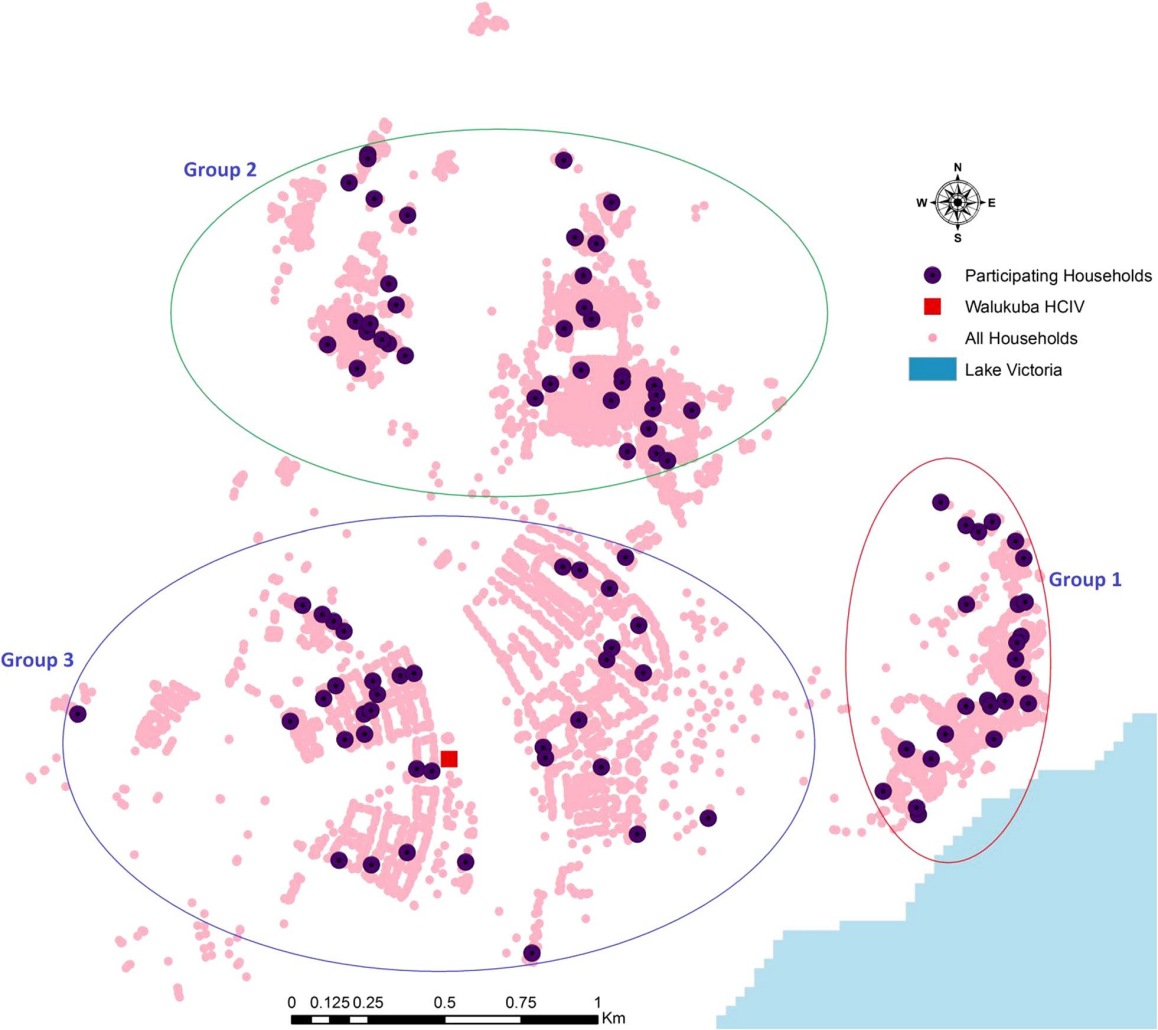
Geographical clustering of participating households, in Walukuba, arbitrarily identified as *Groups 1, 2* and *3*, and these present distinctly varied entomological characteristics

Within the more urban site of Walukuba, individual households with characteristics of greater urbanicity were associated with a lower HDM including a household density >80 per 100 m radius (IRR = 0.30, p < 0.001), NDVI ≤0.45 (IRR = 0.35, p < 0.001), night-time lights >3 units (IRR = 0.32, p < 0.001), and land cover >20 % (IRR = 0.42, p < 0.001) (Table 1). In contrast, within the two rural sites, individual households with characteristics of greater urbanicity were either not significantly associated with HDM (household density or NDVI), outside the range where associations were found in Walukuba (night-time lights) or lacked sufficient variability to be measured (land cover) (Table 1). Within Walukuba, three geographical clusters of households with distinct characteristics were observed (Fig. 3), including: (1) a fishing village comprised of make-shift wooden housing; (2) slam of predominantly mud and wattle house structures mixed with sub-urban household structures randomly situated without obvious general urban planning; and, (3) old, but fairly orderly, factory workers’ estate housing. Across these clusters, the totals of female *Anopheles* collected over the 24-month period were 1256 (53.3 %), 666 (28.2 %) and 436 (18.5 %), respectively.

### Associations between measures of urbanicity and parasite prevalence

In Walukuba (more urban site), individuals living in households with characteristics of greater urbanicity were associated with lower parasite prevalence, but none of these associations reached statistical significance (Table 2). However, a higher composite score was associated with a significantly lower parasite prevalence (OR = 0.44, p = 0.04). In the rural site of Kihihi, individuals living in households with a household density >80 per 100 m radius were associated with a lower parasite prevalence (OR = 0.15, p < 0.001). In the most rural site of Nagongera, only individuals living in households with an NDVI <0.45 could be evaluated and this measure of urbanicity was not significantly associated with parasite prevalence (Table 2).

### Associations between measures of urbanicity and incidence of malaria

None of the measures of urbanicity, either individually or as a composite score, were associated with a lower incidence of malaria in individual study participants at any of the three sites (Table 3). In Nagongera, however, living in households with an NDVI <0.45 was associated with a higher incidence of malaria (IRR = 1.35, p = 0.01).

## Discussion

Evidence for the impact of urbanicity on reduced malaria prevalence over large scales is clear [3, 8] but the interplay between local heterogeneities, differing malaria metrics and different metrics of urbanicity has rarely been explored. Here, these relationships have been assessed for three sites in Uganda with markedly differing malaria ecologies. The data presented show that variations in the HDM, parasite prevalence and the incidence of malaria in the three sub-counties of Uganda correspond to relative measures of urbanicity across the three sites. Within each site, associations were significant between greater urbanicity and a lower HDM and parasite prevalence in the most urban site (Walukuba), but less so for the two rural sites. Moreover, different approaches to measuring urbanicity translated into slightly different relationships with malaria-relevant metrics, indicating that each urbanicity measure captures different aspects that may or may not be driving mosquito and malaria dynamics.

Increased levels of urbanicity, specifically defined by higher household density, lower levels of NDVI, low residential urban land cover and higher night-time light brightness were observed to be strongly associated with reduced HDM at the urban site, Walukuba, but not in the other two more rural sites. For Walukuba, parasite prevalence was not found to be significantly associated with individual urbanicity metrics at the spatial scales examined here, but rather with the composite urbanicity score. However, increased urbanicity as measured by household density does appear to be associated with reduced parasite prevalence in one rural site, Kihihi, but does not in the other.

Additional observations of disease burden across the three sites indicate that the urban site recorded significant declines in malaria incidence over the study duration [16]. With the observed consistent associations between some but not all urban metrics and malaria metrics, the principal group of urban metrics that showed the most consistent associations with reduced malaria metrics included high household density, low NDVI, high night-time light brightness, and low dense urban land cover. These may be good candidates for future use in modelling the impact of urbanicity on malaria, and wider malaria risk mapping in general.

Whereas higher parasite prevalence was found in the more rural sites than Walukuba, consistent with multiple studies that have reported similar trends [8, 24], only household density had significant associations with it in the rural sites. Unlike this study, other studies have reported significant associations between parasite prevalence and urbanization at sub-continental and global scales [2, 3]. These survey data, however, unlike cohort data, tend to go through long compilation processes, come from various sources collected in differing seasons and often with overlapping age groups [25].

Some aspects of this study limit the ability to draw additional conclusions about urbanicity and malaria transmission. With just 100 households per site, sufficient variability may not have been captured to uncover weaker relationships that may have existed, especially within the more rural sites. These households were, however, randomly selected without bias and are thus likely representative of the populations, and the observational buffers used describe an environment shared by a larger number of households. Another notable limitation is that Nagongera and Kihihi are not so different or diverse in terms of urbanicity, and neither can be described as particularly ‘urban’ overall. High-resolution imagery that could better elicit differences between these sites is not readily available. However, malaria metrics between the two are substantially different and hence do provide a useful contrast in transmission. Factors such as agricultural practices may influence the malaria epidemiology of these rural regions far more than any urban-related factors, but analysis of these were beyond the scope of this current paper.

Other measures used to define urbanicity that were not assessed in this study include the principal economic activity of the population. However, in a study of the risk of malaria in these three sites posed by the nature of house of residence, strong associations were found between HDM and type of house or wealth [26]. In further regard to the measures of urbanization, it was not clear what other factor(s) of urbanization are the most relevant for malaria as a benchmark for the expected outcome to set the stage for auxiliary exploratory work. In addition, this study did not account for contextual factors that may be at play in the study sites, such as coverage of malaria control interventions, multiple interventions’ interference and/or influence, climate and climate change, or variability as played out by political and other forces of influence between jurisdictions of the study sites, to mention just a few. Finally, despite the length of follow-up of the cohorts, in this study temporal variations in urban growth versus malaria were not examined.

In view of these limitations, future work may focus on better understanding the impact of urbanization on malaria through expanded analyses to investigate the role played by other contributory factors, such as rainfall, water bodies, temperature, intervention coverage, as well as nuances of malaria policy disparities across administrative units. Considering that other than space, the temporal aspects of public health are important, additional work should examine the impact of the temporal variations in urbanicity and other factors on malaria. Also, given a stronger understanding of the urbanicity influence on malaria, scaling up analyses to generate local and national risk maps would be a valuable effort for guiding malaria and other public health strategic planning. This, with the integration of mathematical transmission models to bolster guidance for planning for public health and malaria control, would be an important next step. Finally, given previous studies on travel and movement patterns in East Africa, and their perceived, as well as reported correlation with urbanization, proposed future work may incorporate these influences to further establish the contribution of travel and movement coupled with urbanization, to the malaria situation.

Given differences in culture/behaviour, population characteristics, socio-economic strata, and environment between urban and rural communities, these results, though exploratory in nature, support the need for more localized approaches to malaria control and treatment, such as urbanicity tailored packaging of strategic planning foci, intervention approaches, impact evaluation, malaria awareness messages and/or public health campaigns. Africa, more than ever, is becoming increasingly urbanized and targeted interventions such as better garbage and drainage management coupled with LSM in urban areas, would likely results in a great depletion of breeding sites. Given the burden of malaria in sub-Saharan Africa, there is a need to understand the broad impacts of urbanicity on malaria at various scales. Whereas previous studies have quantified these impacts chiefly on large scales and used just single metrics for quantifying the relationship between urbanicity and malaria, this study has shown that the situation is much more complex at smaller scales.

## Abbreviations

IRR: incidence rate ratio
CI: 95 % confidence interval
OR: odds ratio
NDVI: normalized difference vegetation index
LLINs: long-lasting insecticidal nets
IRS: indoor residual spraying
IVM: integrated vector management
LSM: laval source management
GPS: global positioning system
CDC: Centers for Disease Control
HDM: household density of mosquitoes
ETM: enhanced thematic map
VIIRS: visible infrared imaging radiometer suite
PY: per person year
